# Metachronous Occurrence of Granular Cell Tumor in Breast Skin and Scalp: Diagnostic Challenging Differentiating Benign from Malignant and a Literature Review

**DOI:** 10.1155/2016/8043183

**Published:** 2016-01-03

**Authors:** Hampar Akkaya, Havva Serap Toru, Ebru Sebnem Ayva, Zulfikar Karabulut, Cicek Durusoy

**Affiliations:** ^1^Department of Pathology, Başkent University School of Medicine, 06490 Ankara, Turkey; ^2^Department of Pathology, Akdeniz University School of Medicine, 07058 Antalya, Turkey; ^3^Department of Surgery, Başkent University School of Medicine, 06490 Ankara, Turkey; ^4^Department of Dermatology, Başkent University School of Medicine, 06490 Ankara, Turkey

## Abstract

Granular cell tumor (GCT) is a Schwann cell related benign neoplasm of soft tissue. GCT is an uncommon entity that occurs in a wide variety of body sites, but it is generally presented in the skin, oral cavity, superficial soft tissue, and respiratory and digestive tracts. Most of the GCTs are benign but clinically and radiologically these may mimic malignancy. Histopathological diagnosis is gold standard for establishing the true nature of the lesion. GCT is most commonly solitary but in about 10% of cases can be multifocal, usually involving various skin and soft tissue sites versus involving various internal sites. Therefore, these can involve skin and soft tissue or submucosa and viscera. GCT is usually benign; however, local recurrence is common due to incomplete removal. Malignant cases are rarely reported in 1-2% of cases. In this study, we report clinical and histopathological findings of a 36-year-old woman with metachronous GCT in breast and scalp. The clinical features raise the question of whether these are metachronous benign GCTs or whether this is establishment of malignant behavior. The aim of this report is to present the histopathological and clinical features of GCT and the diagnostic challenge of differentiating benign from malignant GCT.

## 1. Introduction

Granular cell tumor, first described in 1926, was thought to be derived from skeletal muscle cells and termed “granular cell myoblastoma” by Abrikossoff [1]. Nowadays, the most accepted theory is Schwann cell origin, because of the S100 protein expression in tumor cells and the similarities between the ultrastructural features of the tumor cells and Schwann cells [2].

GCT is an uncommon tumor that occurs in a wide variety of body sites but is mainly found in the skin, oral cavity, superficial soft tissue, and respiratory and digestive tracts [3]. GCT of the breast is really rare. It accounts for 8.5% of all GCTs [4]. GCT arises from interlobular breast stroma or cutaneous tissue of breast [1, 2, 5]. GCT is usually benign. Malignant cases are rarely reported, with an incidence of 1-2% [6]. Recognition of benign GCT is important, since these are infrequently diagnosed preoperatively; these may be confused clinically and radiologically with malignant lesions.

Herein we report the clinical and histopathological findings in a case of a 36-year-old woman with a clinical presentation of metachronous GCTs, a palpable, painless mass in the breast skin, and then another mass eighteen months later in the scalp. A literature review is performed and discussion between benign and malignant GCT is presented.

## 2. Case Report

**Figure 1:**
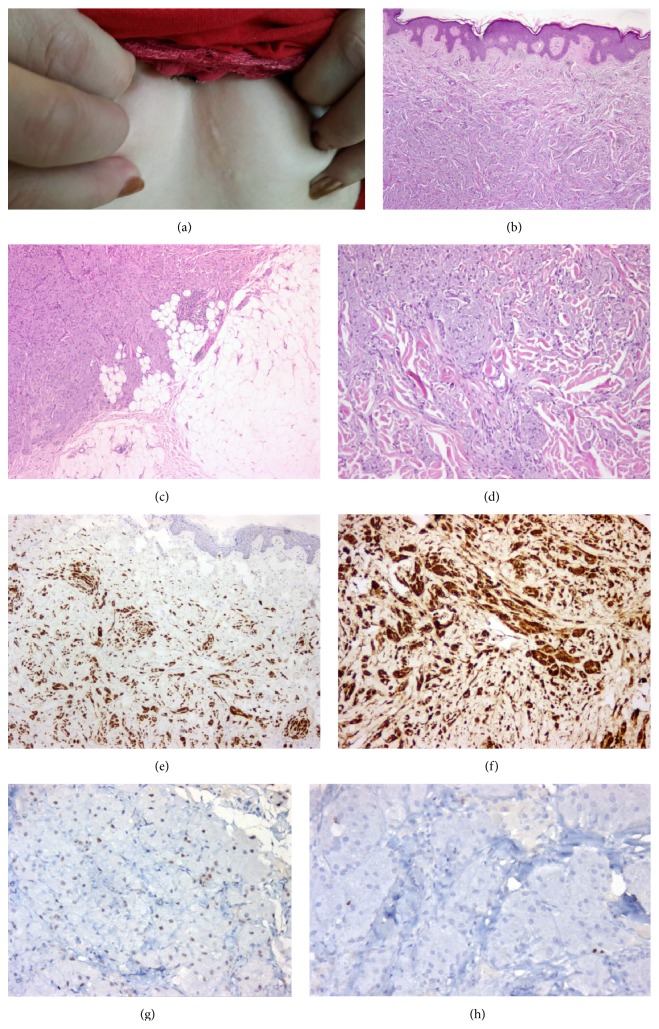
(a) Subcutaneous mass of breast. (b) Minimal acanthosis and epidermal pseudoepitheliomatous hyperplasia (H&E, ×50). (c) Ill-defined tumoral lesion with infiltrative pattern (H&E, ×100). (d) Compact nests and sheets of polygonal tumor cells (H&E, ×200). (e) Tumor cells expressing S100 (×100). (f) Tumor cells expressing CD68 (×200). (g) Tumor cells weakly expressing p53 (×200). (h) Ki-67 proliferation expressing approximately 1%.

*Case 1.* A 36-year-old woman presented with a firm, fixed, painless palpable skin mass, approximately measured 2 cm diameter, in the lower-inner quadrant of the left breast skin (Figure 1(a)). She had pruritus but had neither nipple discharge, nor peau d'orange appearance. A slowly growing mass was realized a few years ago. The patient had no previous surgical intervention of the breast. Macroscopically an ill-defined, firm, grey-white lesion measured approximately 1.6 × 0.9 × 0.9 cm in the subepidermal breast tissue. Histopathologically, this lesion was located in the dermis and subcutaneous fat tissue without epidermal infiltration. Minimal acanthosis without reactive pseudoepitheliomatous hyperplasia was detected (Figure 1(b)). The tumoral lesion was ill-defined and had an infiltrative growth pattern but the ductal-lobular unit of the breast was not involved (Figure 1(c)). The lesion was composed of compact nests and sheets of large polygonal tumoral cells, containing large eosinophilic granular cytoplasm and relatively small round or oval nuclei (Figure 1(d)). Focally, small nerves were surrounded by granular cells. Tumor cells were expressing S100 protein strongly and diffusely (Figure 1(e)) and CD68 in cytoplasmic inclusions surrounded by halos (Figure 1(f)) and were completely negative for p53 (Figure 1(g)), pancytokeratin (cytokeratin AE1/AE3), estrogen receptor, and progesterone receptor. Ki-67 labeling index of tumor was approximately 1% (Figure 1(h)). 

**Figure 2:**
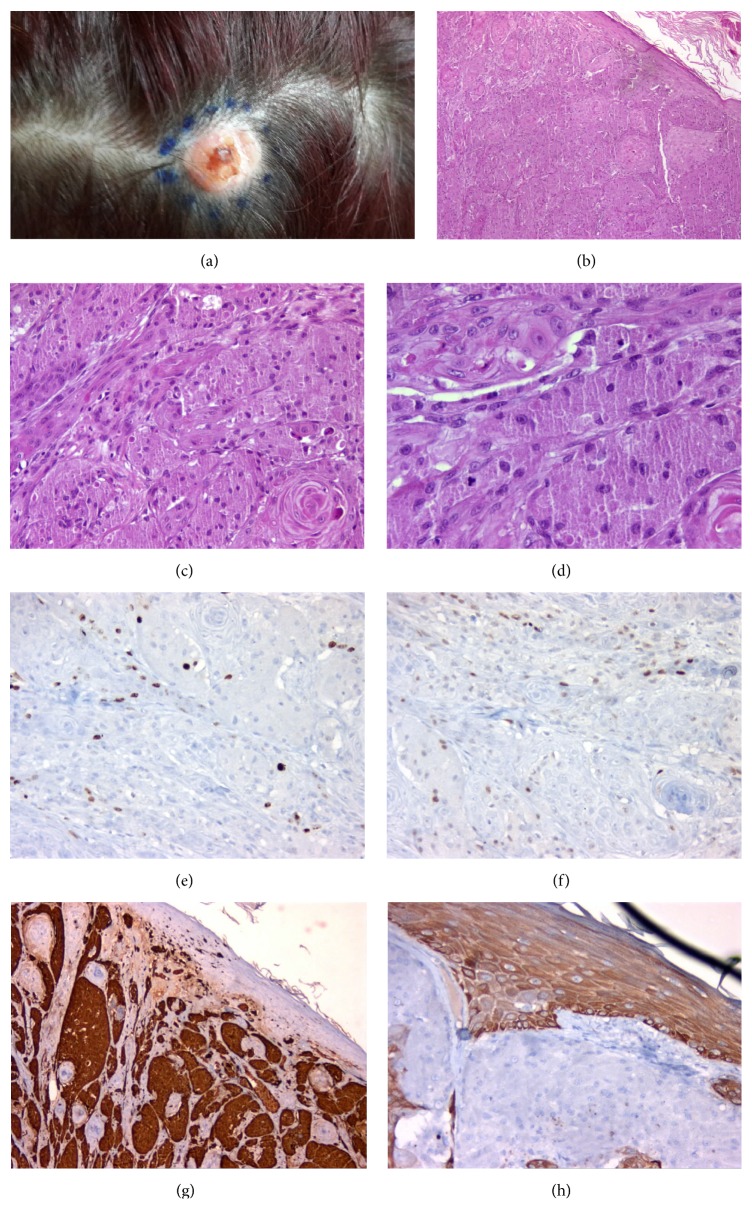
(a) Subcutaneous mass on the vertex of the scalp with ulcerated surface. (b) Reactive pseudoepitheliomatous hyperplasia of epidermis (H&E, ×100). (c) Polygonal tumor cells forming nests (×200). (d) Tumor cells with large eosinophilic granular cytoplasm and oval nucleus with nucleoli (H&E, ×400). (e) Tumor cells showing approximately 6% Ki-67 proliferation index (×200). (f) Tumor cells expressing p53 weakly and mildly (×200). (g) Tumor cells expressing S100 protein diffusely and strongly. (h) Tumor cells immunohistochemically negative for cytokeratin AE1/AE3 (×200).

*Case 2*. Eighteen months later, the patient presented with a mass on the vertex of scalp (Figure 2(a)). This lesion was 1.7 × 1.3 × 0.8 cm in size and with scale-crusted appearance. This tumoral lesion was located in dermis and was well defined macroscopically and microscopically, in contrast to breast lesion. We observed prominent reactive pseudoepitheliomatous hyperplasia in the scalp lesion (Figure 2(b)). This lesion had similar histological, cytomorphological, and immunohistochemical features to the previous lesion (Figures 2(c) and 2(d)); Ki-67 was relatively overexpressed in the scalp lesion (6% of the nuclei of neoplastic cells) (Figure 2(e)). Tumors cell expressed weak and mild p53 (Figure 2(f)). Similar to previous lesion tumor cells were diffusely positive for S100 (Figure 2(g)) and negative for pancytokeratin (cytokeratin AE1/AE3) (Figure 2(h)).

In both of the lesions none of the histological criteria of malignant behavior was detected such as (1) spindling of the tumor cells, (2) presence of vesicular nuclei with large nucleoli, (3) increased mitotic rate, (4) high nuclear to cytoplasmic ratio, (5) pleomorphism, or (6) necrosis.

## 3. Discussion

GCT was first described in 1926 by Abrikossoff on tongue and postulated a myogenic origin as “granular cell myoblastoma.” Initially GCTs were considered to arise from myocytes, histiocytes, fibroblast, or intestinal mesenchymal cells. The most widely accepted theory has been that of Schwann cell origin, because of positivity of the tumor cells for the S100 protein and the similarities between the ultrastructural features of the tumor cells and those of Schwann cells.

GCT occurs in patients of all ages, commonly observed between the fourth and sixth decades of life. It is more common in women than men and very rare in childhood [7–9].

GCT arises throughout the body and the most common presentation is a solitary painless nodule, located in the skin, tongue, and oral cavity and less frequently in breast, gastrointestinal and respiratory tracts, female genital system, smooth muscle, or striated muscle [9]. According to the literature, 30% to 45% of GCTs are observed in the skin of the head and neck. When compared to other studies, Dupuis and Coard found a notable difference in the distribution of GCT. In their study, lesions of the tongue accounted for fewer than expected, while lesions of the breast and vulva were considerably increased [8].

GCT of the breast is rare, with a percentage of 8.5 among all GCTs [10, 11]. Breast GCT more commonly occurs in premenopausal women and, according to reports, especially in African-American women [2, 12]. GCT is found most frequently in the upper inner quadrant of the breast, differentiating it from carcinomas which is frequently in the outer quadrants. In our case, the GCT tumor was located in the lower inner quadrant of the left breast as a dermal infiltration.

GCTs are usually benign solitary Schwannian neural tumors. Local recurrence may occur with incomplete removal. Malignant behavior of GCT is rare (<2%). Although most of GCTs are solitary lesions, in about 15 to 25% of the cases GCT can be multifocal, simultaneously involving the skin and soft tissue or simultaneously involving the submucosa and viscera. Multiplicity should not be taken as evidence for malignancy [13]. Familial occurrence has been reported, but there is no clear evidence for a syndrome [14].

Malignant GCTs usually occur in older population and tend to be larger than their benign counterparts. Malignant GCTs grow rapidly, often ulcerate, invade locally, and tend to spread with extensive metastases. Generally, it is well accepted that benign GCTs do not transform into malignant ones; malignant GCTs arise de novo. In contrast, Chen et al. presumed that malign GCT may result from the malignant transformation of benign GCT [15]. Malignant GCTs often recur locally with multiple skin satellite nodules. Metastases most commonly develop in the lymph nodes, lungs, bones, rarely the intestines, liver, or brain. According to Fanburg-Smith et al., tumor-related deaths occurred in 11 of 28 patients, with a median time of 3 years (range, 1 yr–9 yr) [16].

Grossly, benign GCT typically forms an oval nodule, smaller than 3 cm, that varies from being well or poorly circumscribed with a pale and yellow tan on cut surface. Occasionally, tumors reach the size of 5 cm or more. Dupuis and Coard reported the range of diameter as 0.2 cm to 10 cm in 130 benign GCT cases of various sites [8]. Malignant GCTs have similar gross appearance but tend to be larger than benign lesions; however, malignant GCT can also be small. Microscopically most of malignant GCTs are poorly circumscribed. About two-thirds of the GCTs are located in cutaneous, subcutaneous, or submucosal tissues and some of these cases are associated with pseudoepitheliomatous hyperplasia of the overlying epidermis or mucosal epithelium. It is reported that GCT of breast may arise in either interlobular breast stroma or the skin overlying breast [1, 2, 11]. The cells of GCT which are round, polygonal, or slightly spindle shaped and are characterized by large and granular cytoplasm. They have small and hyperchromatic or large and vesicular nuclei. Granular cells and peripheral nerves have close association. In GCT, there are small nerves that are invaded or surrounded by the granular cell clusters.

Immunohistochemically, GCT is consistently positive for S100 protein, NSE, various myelin proteins, and negative for muscle cell and epithelial markers. GCTs are strongly positive for macrophage antigen CD68, specifically in the cytoplasmic lysosomal granules with clear halo around these, and this lysosomal granule excludes other tumors that may have granular features. GCTs are also alpha-inhibin positive [17–19].

In 1998, Fanburg-Smith et al. established that six histological criteria could predict malignant behavior [16], such as sarcomatoid spindling of the tumor cells, presence of vesicular nuclei with large nucleoli, increased mitotic rate (2 mitoses per 10 high-power fields), high nuclear to cytoplasmic ratio, pleomorphism, and necrosis. If a GCT demonstrates three or more of these criteria, it is classified as “malignant” and those that show one or two criteria are classified as “atypical,” and if it exhibits none of the criteria or only focal pleomorphism, it is classified as “benign.” Most of the malignant tumors, however, had at least 5 or 6 of the criteria and most malignant cases had necrosis or increased mitotic activity. They also demonstrated that Ki-67 and p53 were significantly higher in atypical and malignant tumors than in benign ones [16].

According to histological and cytological features, Argenyi suggested that malignant GCTs might be classified into two types [20]. The first type of malignant GCT is more common and essentially appears identically to a benign tumor; because cytologic atypia or mitotic activity may not be reliable biologic indicators in this form, diagnosis of malignancy should be made by clinicopathologic correlation (large size, rapid growth, ulceration, necrosis, sarcomatoid spindling, and lymphatic and vascular invasion). Vesicular nuclei with large nucleoli and mitotic rate are more than 2 mitoses/10 HPF and are additional findings for malignancy. The second type of malignant GCT is quite rare. In this type, either primary GCT or metastases display conventional malignant histological and cytological features. Malignant GCT cells express similar immunohistochemical features to those of a benign tumor, with the exception Ki-67 labeling index being higher and p53 expression being prominent [20].

According to Nasser et al., some of the criteria proposed by Fanburg-Smith, such as pleomorphism and increased nuclear-to-cytoplasmic ratio, are subject to interobserver variation and show weak reproducibility among different pathologists, complicating the diagnostic spectrum [21]. It is thought that simple, practical, and clear diagnostic criteria are required. In 2011, Nasser et al. classified the GCTs based on the presence of necrosis (whether single cell or en masse) and mitoses. They believe that these are two of the most reliable and more reproducible criteria associated with malignancy and designated tumors without these 2 features as “benign” GCTs and those cases demonstrating at least one of the previously mentioned features as GCTs with “uncertain malignant potential.” They considered metastases as the only definitive criterion of malignancy, regardless of the histopathologic features [21]. Their paper did not have the large number of cases as in the AFIP paper, but their conclusions were similar, overall.

p53 expression has been described in neoplastic cells of MGCTs with a variable frequency, ranging from 5% to 100% [15, 16, 22, 23]. Ki-67 proliferating index ranged from 10% to 50% [16, 19, 21, 23]. Le et al. reported that there was not any significant difference between benign and atypical cases' Ki-67 expression [19]. Nasser et al. reported mean of Ki-67 labeling index 10.5% in atypical GCTs and 2.7% in benign GCTs and the difference was statistically significant. There were no significant differences in p53 expression between three groups; they all showed strong and diffuse nuclear staining [21].

Essentially any tumor type may show granular cell change, and thus the differential diagnosis of true granular cell tumors is quite broad. Similar morphological properties can be observed in smooth muscle tumors, rhabdomyoma, hibernoma, fibroxanthoma, and malignant melanoma [24]. GCT is distinguished from other granular cell lesions with S100 protein positivity. Epithelial tumors are separated from granular cell features with keratin positivity and S100 protein negativity. GCTs of the breast must be distinguished from benign and malign tumors. Histiocytes and granular cells are similar in appearance, so that histiocytic inflammatory lesions must be differentiated. Carcinomas of breast with apocrine or histiocytoid features may resemble GCT. Carcinomas are positive for cytokeratin, and DCIS is usually present in breast carcinomas. Malignant tumors that metastasize to breast like malignant melanoma, renal cell carcinoma must be distinguished from GCT. The most challenging tumors in differential diagnosis of GCT are granular cell (spindle cell) melanoma, which is lack of cytoplasmic inclusions with halos and, when spindled, some degree of cytologic atypia and prominent nucleoli and lymphoid response may be present. BRAF can also be helpful in this setting.

In our case, the patient was a 36-year-old woman. The first lesion was in lower inner quadrant of the left breast; the second lesion occurring 18 months later was on the vertex of the scalp.

In immunohistochemical examination of p53, both lesions were similar (approximately 80% weak nuclear positivity). Ki-67 labeling index was 1% in the breast GCT and 6% in scalp GCT. Ki-67 expression was slightly higher in scalp GCT than breast GCT. Another important point was evaluating Ki-67 away from pseudoepitheliomatous hyperplasia, because basal layer of these areas may overexpress Ki-67. Because of this, we evaluated the Ki-67 labeling index areas of tumor away from epidermis with pseudoepitheliomatous hyperplasia.

After microscopic and immunohistochemical examination, both tumors were diagnosed as benign and metachronous according to the diagnostic criteria stated above [16, 21].

Tumor cells are located in dermis and extend to the subcutaneous septa. Due to subcutaneous septal infiltration and perineural spread, characteristic findings of the GCT, incomplete removal, and local recurrence are common problems. Therefore, complete excision of GCT is important. After complete surgical resection of both tumors with safe surgical margins, patients could be clinically followed.
